# Cordycepin Induces Apoptosis through JNK-Mediated Caspase Activation in Human OEC-M1 Oral Cancer Cells

**DOI:** 10.1155/2022/1842363

**Published:** 2022-10-03

**Authors:** Kuo-Lung Tung, Su-Zhen Wu, Chun-Chuan Yang, Hong-Yi Chang, Chun-Sheng Chang, Yan-Hsiung Wang, Bu-Miin Huang, Yu-Yan Lan

**Affiliations:** ^1^Department of Oral Hygiene, Shu-Zen Junior College of Medicine and Management, Kaohsiung 82144, Taiwan; ^2^School of Dentistry, College of Dental Medicine, Kaohsiung Medical University, Kaohsiung 80708, Taiwan; ^3^Department of Anesthesia, Chi-Mei Medical Center, Liouying, Tainan 73657, Taiwan; ^4^Department of Nursing, Min-Hwei Junior College of Health Care Management, Tainan 73658, Taiwan; ^5^Department of Dental Technology, Shu-Zen Junior College of Medicine and Management, Kaohsiung 82144, Taiwan; ^6^Department of Biotechnology and Food Technology, College of Engineering, Southern Taiwan University of Science and Technology, Tainan 71005, Taiwan; ^7^Orthopaedic Research Center, College of Medicine, Kaohsiung Medical University, Kaohsiung 80708, Taiwan; ^8^Regenerative Medicine and Cell Therapy Research Center, Kaohsiung Medical University, Kaohsiung 80708, Taiwan; ^9^Department of Medical Research, Kaohsiung Medical University Hospital, Kaohsiung Medical University, Kaohsiung 80708, Taiwan; ^10^Department of Medical Research, China Medical University Hospital, China Medical University, Taichung 40402, Taiwan; ^11^Department of Cell Biology and Anatomy, College of Medicine, National Cheng Kung University, Tainan 70101, Taiwan; ^12^School of Medicine, College of Medicine, I-Shou University, Kaohsiung 82445, Taiwan

## Abstract

Cordycepin, a bioactive compound extracted from *Cordyceps sinensis*, can induce apoptosis in human OEC-M1 oral cancer cells. However, the exact mechanism is still unclear. The present study aimed to investigate the underlying mechanism of cordycepin-induced apoptosis in OEC-M1 cells. Following treatment with cordycepin, apoptosis was examined and quantified using a DNA laddering assay and a cytokeratin 18 fragment enzyme-linked immunosorbent assay, respectively. Expressions of mitogen-activated protein kinases (MAPKs) and apoptosis-related proteins were detected by the western blot analysis. Our results show that a pan-caspase inhibitor, Z-VAD-FMK, could significantly inhibit cordycepin-induced apoptosis in OEC-M1 cells. In addition, treatment with cordycepin not only activated caspase-8, caspase-9, and caspase-3 but also induced Bid and poly ADP-ribose polymerase cleavages. Furthermore, cordycepin also induced the activation of c-Jun N-terminal kinase (JNK), extracellular signal-regulated kinase, and p38 MAPKs. Among MAPKs, activation of JNK solely contributed to cordycepin-induced apoptosis with the activation of caspase-8, caspase-9, and caspase-3 and cleavage of PARP. Taken together, the present study demonstrated that cordycepin activated JNK and caspase pathways to induce apoptosis in OEC-M1 cells.

## 1. Introduction

Oral cancer is a subgroup of squamous cell carcinoma of the head and neck, presenting on the lips, mouth, and upper throat around the oral cavity. High-risk factors for developing oral cancers include tobacco use, betel quid chewing, alcohol consumption, and oral chronic inflammation [1, 2]. With a high incidence, oral cancer has been a public health problem in India, Sri Lanka, Thailand, South China, and Taiwan [3]. Although advances have been made in clinical treatments, the 5-year survival rate of oral cancer patients after surgery and radiotherapy treatments remains low, at about 50%, and has not been improving in the past few decades [4]. Thus, there is an urgent and large-scale need for novel, effective drugs to improve the prognosis of oral cancer patients.

Recent studies have shown that extracts from certain plants in the wild have high application value in antiobesity and diabetes therapy, neuromodulation, antivirus activity, and nanomaterial synthesis [5–9]. Plant extracts have also been used to fight cancers. For instance, certain small molecules from natural sources have been used to treat breast cancer [10]. Another example is curcumin, a natural bioactive compound extracted from *Curcuma longa*, which was shown to induce apoptosis of cancer cells and suppress tumor growth [11–13]. Yet another, paclitaxel, a substance extracted from the Pacific yew tree, *Taxus brevifolia*, is clinically used to treat various cancers [14].

In this study, we have focused on cordycepin, a bioactive compound extracted from *Cordyceps sinensis*, which has multiple documented pharmacological activities in human and animal models, including immunomodulatory [15], anti-inflammatory [16], and hypoglycemic effects [17]. Furthermore, several studies have demonstrated that cordycepin not only has the ability to induce apoptosis in various cancer cell lines *in vitro* [18–20] but also suppresses tumor growth *in vivo* [18, 19], suggesting that the antitumor effects of cordycepin are related to apoptosis.

Apoptosis is a form of programmed cell death. Apoptotic cells are characterized by cell shrinkage, plasma membrane blebbing, apoptotic body formation, chromatin condensation, cytokeratin 18 cleavage, and DNA fragmentation [21–23]. The two major pathways of apoptosis are the death receptor (extrinsic) pathway and the mitochondrial (intrinsic) pathways, with the initiator caspase protein and effector caspase protein playing important roles within apoptotic pathways [21–23]. For instance, initiator caspase-8 and caspase-9 are the mediators of the death receptor pathway and mitochondrial pathway, respectively [21–23]. Once these initiator caspases are activated, they promote the activation of downstream effector caspases (caspase-3, caspase-6, and caspase-7), which lead to the cleavage of Lamin and poly ADP-ribose polymerase (PARP), and finally, to apoptosis [22].

Mitogen-activated protein kinases (MAPKs), a family of serine and threonine protein kinases, include as its most important subfamilies extracellular signal-regulated kinases (ERKs), c-Jun N-terminal kinases (JNKs), and p38 MAPKs [24]. MAPKs are known to promote caspase cascades during apoptosis [25–28]. For instance, ERK is required for caspase-3 activation in cisplatin-induced apoptosis in HeLa cells [25]. However, JNK has been reported to contribute to caspase-9 and caspase-3 activations under gemcitabine treatment in human lung cancer H1299 cells [26]. Finally, p38 MAPK is associated with caspase-8 activation in TGF*β*-mediated apoptosis in human Burkitt lymphoma B cells BL41 [27].

We reported earlier that cordycepin could induce apoptosis in human OEC-M1 oral cancer cells [29]. However, the mechanism involved in the cordycepin-induced apoptotic effects remains to be elucidated. Here we investigated those mechanisms in OEC-M1 oral cancer cells because the insights thus gained have great therapeutic potential for oral cancer. The results of these experiments revealed that cordycepin activated JNK and caspase pathways to induce apoptosis in OEC-M1 cells. Our findings can be used to design more successful therapy regimes combined with cordycepin anticancer effects to improve the poor prognosis of patients with oral cancers.

## 2. Materials and Methods

### 2.1. Materials

Cordycepin, penicillin, streptomycin, dimethyl sulfoxide, bovine serum albumin, HEPES, SP600125, SB202190, and sodium bicarbonate (NaHCO_3_) were purchased from Sigma-Aldrich (MN, USA); PD184352, from Enzo Life Sciences (Farmingdale, NY, USA); fetal bovine serum (FBS) and RPMI 1640 medium, made by Invitrogen/Thermo Fisher Scientific (Waltham, MA, USA); and JNK6o and Z-VAD-FMK, from R&D Systems (Minneapolis, MN, USA).

### 2.2. Cell Lines and Cultures

OEC-M1 cells, derived from gingival epidermal carcinoma, and OC3 cells, derived from buccal epidermal carcinoma, were obtained from Prof. Kuo-Wei Chang (National Yang-Ming University, Taipei, Taiwan). OEC-M1 cells were cultured in RPMI-1640 medium supplemented with 10% FBS, 24 mM NaHCO_3_, 25 mM HEPES, 100 U/ml penicillin, and 100 *μ*g/ml streptomycin. OC3 cells were cultured in a 1 : 1 mixture of DMEM and keratinocyte serum-free medium supplemented with 10% FBS. Both cell lines were incubated in a humidified incubator at 37°C with 5% CO_2._

### 2.3. Enzyme-Linked Immunosorbent Assay (ELISA)

Cytokeratin 18 fragment levels in the cell culture supernatants were quantified by using the SimpleStep ELISA kits (Cat. No. ab254515; Abcam, Plc., Cambridge, UK) according to the manufacturer's instructions.

### 2.4. Western Blotting

The preparation of cell lysate and western blotting were carried out as described previously [29]. Primary antibodies, including rabbit antibodies against phospho-JNK (cat. no. 9251; 1 : 1000), total JNK (cat. no. 9252; 1 : 2000), phospho-ERK (cat. no. 9101; 1 : 1000), total ERK (cat. no. 9102; 1 : 2000), phospho-p38 MAPK (cat. no. 9215; 1 : 1000), total p38 MAPK (cat. no. 9212; 1 : 2000), cleaved caspase-8 (cat. no. 9496; 1 : 1000), cleaved caspase-9 (cat. no. 7237; 1 : 1000), cleaved caspase-3 (cat. no. 9662; 1 : 1000), and cleaved PARP (cat. no. 9532; 1 : 1000) and mouse antibodies against total Bid (cat. no. 8762; 1 : 1000) and *β*-actin (cat. no. 58169; 1 : 5000), were purchased from Cell Signaling Technology (MA, USA). The secondary antibodies used in the assay, including horseradish peroxidase (HRP)-conjugated goat anti-rabbit IgG (cat. no. 111-035-144; 1 : 5,000) and HRP-conjugated goat anti-mouse IgG (cat. no. 111-035-146; 1 : 5,000), were purchased from Jackson Immuno Research (PA, USA). The optical density of protein bands was quantified by using Quantity One (PDI, Huntington Station, NY, USA), a computer-assisted image analysis system.

### 2.5. DNA Ladder Assay

DNA fragmentation, a hallmark of apoptosis, was detected by using a DNA Laddering kit (Cat. No. 660990; Cayman Chemical, Ann Arbor, MI, USA) according to the manufacturer's instructions.

### 2.6. Statistical Analysis

Statistical analyses were performed by using SPSS version 17.0 software (SPSS, Inc., Chicago, IL, USA). All experiments were performed in triplicate, and the data are presented as the mean ± standard error of the mean. The significance of differences between two groups was evaluated with the unpaired *t*-test; differences between multiple groups were evaluated with the one-way ANOVA and Tukey's post hoc test. Statistical significance was set at *p* < 0.05.

## 3. Results

### 3.1. Cordycepin Induces Apoptotic Effects in OEC-M1 Cells through Activation of Caspases

**Figure 1:**
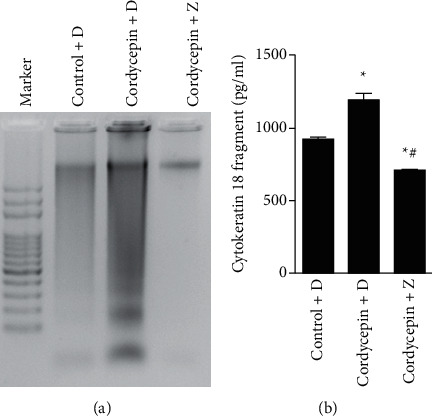
Cordycepin induces apoptotic effects in OEC-M1 cells through the activation of caspases. OEC-M1 cells were treated without (control) or with 100 *μ*M cordycepin and concurrently treated with the solvent control DMSO (D) or a 10 *μ*M Z-VAD-FMK, an irreversible pan-caspase inhibitor (Z). (a) DNA fragmentation was assessed by using the DNA laddering assay. (b) Cytokeratin 18 fragment concentration in the cell culture supernatants was determined by ELISA. ^*∗*^*p* < 0.05 vs. control + DMSO group; ^#^*p* < 0.05 vs. cordycepin + DMSO group.

We have previously shown that cordycepin could induce apoptosis in OEC-M1 cells [29]. Given that caspase cascades play critical roles in mediating apoptotic cell death [22], we first examined whether caspases are involved in cordycepin-induced apoptosis in OEC-M1 cells.  Figure 1(a) shows that treatment with Z-VAD-FMK, a pan-caspase inhibitor that can inhibit caspase activation, could block cordycepin-induced DNA fragmentation. It has been shown that caspase-cleaved cytokeratin 18, a biochemical marker of apoptosis, is released from epithelial cells during apoptosis [23]. The analysis by ELISA showed that cordycepin boosted the amount of cytokeratin 18 fragments released by OEC-M1 cells significantly above control levels (Figure 1(b), *p* < 0.05), but this effect was fully reversed by treatment with the caspase inhibitor Z-VAD-FMK (Figure 1(b), *p* < 0.05). These results indicate that the activation of caspases is essential for cordycepin-induced apoptosis in OEC-M1 cells.

### 3.2. Cordycepin Induces Activation of Caspase-8, Caspase-9, Caspase-3, and PARP Cleavage in OEC-M1 Cells

**Figure 2:**
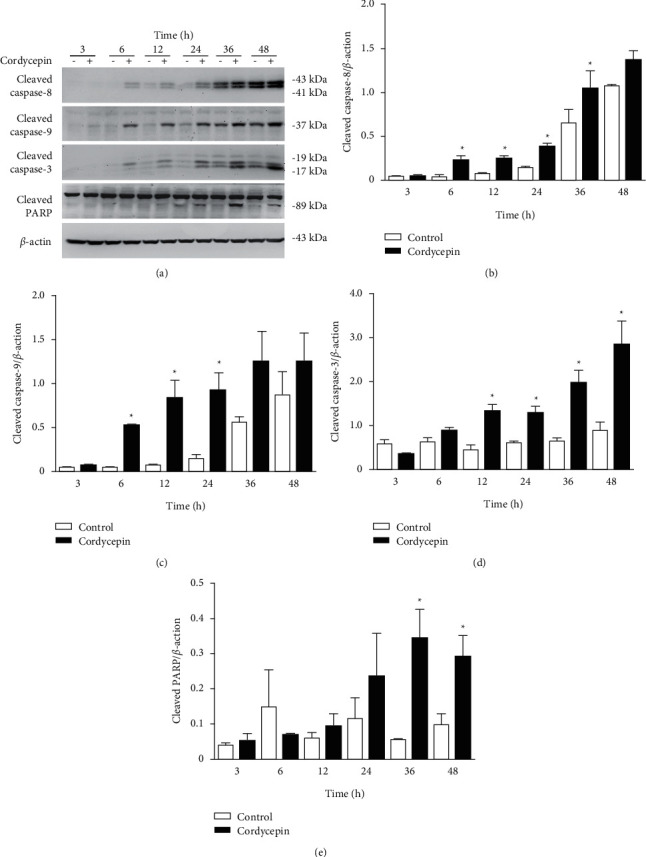
Cordycepin induces activation of caspase-8, caspase-9, caspase-3, and PARP cleavage in OEC-M1 cells. (a) OEC-M1 cells were treated without (-) or with 100 *μ*M cordycepin (+) for 3, 6, 12, 24, 36, and 48 h, respectively. Protein expression of cleaved caspase-8, cleaved caspase-9, cleaved caspase-3, cleaved PARP, and *β*-actin (internal control) was visualized using western blotting. Protein levels in the blots were quantified using the Quantity One image analysis system for cleaved (b) caspase-8, (c) caspase-9, (d) caspase-3, (e) and PARP. ^*∗*^*p* < 0.05 vs. control.

Proteolytic cleavage activates initiator caspases (caspase-8 through the death receptor pathway and caspase-9 through the mitochondrial pathway) [22], which initiate a proteolytic cascade to activate downstream caspases, such as caspase-3, eventually leading to the cleavage of various cellular substrates involved in apoptosis [22]. To investigate caspase activation by cordycepin, we treated OEC-M1 cells with cordycepin for a variable duration (3, 6, 12, 24, 36, and 48 h) and used western blotting to monitor the amount of cleaved caspases (activated caspases) released by the cells after treatment (Figure 2(a)). The quantitative analysis of the blots revealed that caspase-8 was activated significantly above the control levels at 6, 12, 24, and 36 h post-treatment (Figure 2(b), *p* < 0.05). Similarly, caspase-9 was significantly activated at 6, 12, and 24 h post-treatment (Figure 2(c), *p* < 0.05), and caspase-3 was significantly activated at 12, 24, 36, and 48 h post-treatment (Figure 2(d), *p* < 0.05). We also assayed PARP, a DNA repair enzyme and a hallmark of apoptosis, as it is known to be cleaved by effector caspases during apoptosis [22]. Western blotting provided evidence of significant expression of cleaved PARP at 36 and 48 h after cordycepin treatments of OEC-M1 cells (Figure 2(e), *p* < 0.05).

### 3.3. Cordycepin Induces Bid Cleavage in OEC-M1 Cells

**Figure 3:**
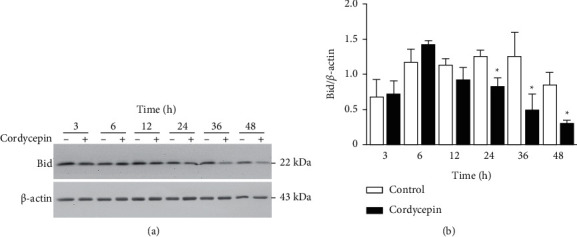
Cordycepin induces Bid cleavage in OEC-M1 cells. (a) OEC-M1 cells were treated without (-) or with 100 *μ*M cordycepin (+) for 3, 6, 12, 24, 36, and 48 h, respectively. Protein expression of Bid and *β*-actin (internal control) was examined using western blotting. (b) The Bid expression, quantified by using the Quantity One image analysis system, declined over time due to cleavage induced by cordycepin. ^*∗*^*p* < 0.05 vs. control.

The Bid is a proapoptotic protein in the Bcl-2 family, downstream of caspase-8. Caspase-8 activates Bid by cleaving it into t-Bid, which leads to the initiation of the mitochondrial pathway [22]. After demonstrating that caspase-8 was activated by cordycepin, we next used western blotting to determine whether Bid is cleaved in cordycepin-treated OEC-M1 cells (Figure 3(a)). The quantitative analysis of the blots shows that cleavage of Bid significantly occurred at 24, 36, and 48 h treatments of cordycepin (Figure 3(b), *p* < 0.05).

### 3.4. Cordycepin Activates JNK, ERK, and p38 MAPK in OEC-M1 Cells

**Figure 4:**
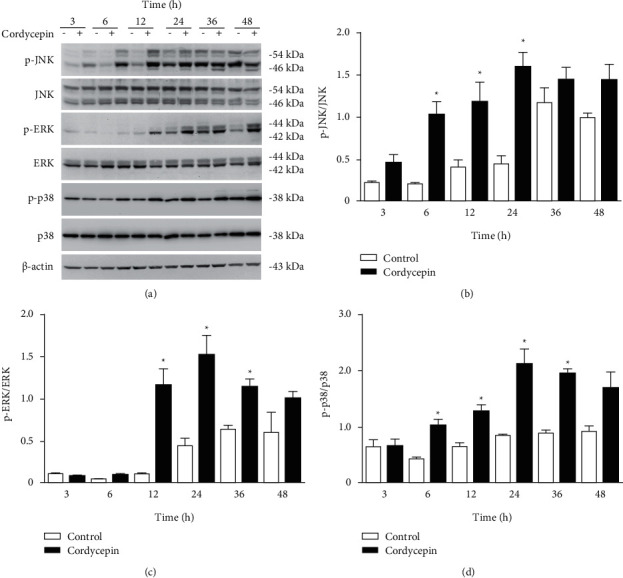
Cordycepin activates JNK, ERK, and p38 MAPK in OEC-M1 cells. (a) OEC-M1 cells were treated without (-) or with 100 *μ*M cordycepin (+) for 3, 6, 12, 24, 36, and 48 h, respectively. Protein expression levels were visualized using western blotting for phosphorylated JNK, total JNK, phosphorylated ERK, total ERK, phosphorylated p38 MAPK, total p38 MAPK, and *β*-actin (internal control). Protein expression levels were quantified using the Quantity One image analysis system for (b) activated JNK, (c) activated ERK, and (d) activated p38 MAPK. ^*∗*^*p* < 0.05 vs. control.

The MAPKs family consists of JNK, ERK, and p38 MAPK proteins, each of which plays an essential role in activating caspases during apoptosis [25–28]. To determine which of the MAPKs proteins were triggered by cordycepin treatment, we treated OEC-M1 cells with cordycepin for 3, 6, 12, 24, 36, and 48 h, respectively, and monitored protein expression for activated (phosphorylated) and total MAPK proteins using western blotting (Figure 4(a)). The results of the quantitative analysis revealed that JNK was significantly activated in OEC-M1 cells at 6, 12, and 24 h after cordycepin treatment (Figure 4(b), *p* < 0.05). Similarly, ERK was significantly activated at 12, 24, and 36 h after treatment (Figure 4(c), *p* < 0.05) and p38 MAPK was significantly activated at 6, 12, 24, and 36 h post-treatment (Figure 4(d), *p* < 0.05).

### 3.5. Activation of JNK Is Required for Cordycepin-Induced Apoptosis in Oral Cancer Cells

**Figure 5:**
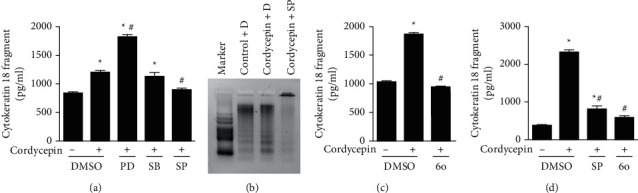
Activation of JNK is required for cordycepin-induced apoptosis in oral cancer cells. The concentration of cytokeratin 18 fragments (an apoptosis marker) in the cell culture supernatants was determined by ELISA. (a) OEC-M1 cells were treated without (-) or with 100 *μ*M cordycepin (+) and concurrently treated with either 5 *μ*M SP600125 (pan-selective JNK inhibitors) or 5 *μ*M PD184352 (selective ERK inhibitor) or 5 *μ*M SB202190 (selective p38 MAPK inhibitor) or a solvent control (DMSO), for 48 h. (b) OEC-M1 cells were treated without (control) or with 100 *μ*M cordycepin and concurrently treated with 5 *μ*M SP600125 (SP) or the solvent control DMSO (D) for 48 h; DNA fragmentation was assessed by using the DNA laddering assay. (c) OEC-M1 cells were treated without (-) or with 100 *μ*M cordycepin (+) and concurrently treated with 5 *μ*M JNK6o (6o), or a solvent control, DMSO, for 48 h. (d) OC3 cells were treated without (-) or with 100 *μ*M cordycepin (+) and concurrently treated with 5 *μ*M SP600125 (SP), 5 *μ*M JNK6o (6o), or a solvent control, DMSO, for 48 h. ^*∗*^*p* < 0.05 vs. control + DMSO group; ^#^*p* < 0.05 vs. cordycepin + DMSO group.

The above experiments demonstrated that cordycepin-activated JNK, ERK, and p38 MAPK were present in OEC-M1 cells (Figure 4). Next, we used selective inhibitors of JNK (SP600125), ERK (PD184352), and p38 MAPK (SB202190), to determine which of these MAPKs was the major mediator of cordycepin-induced apoptosis in OEC-M1 cells. We found that, among the inhibitors, only the JNK inhibitor SP600125 could remarkably reduce the cordycepin-induced release of cytokeratin 18 fragments, a marker of apoptosis (Figure 5(a), *p* < 0.05). We also verified the role of JNK activation in cordycepin-induced apoptosis in an independent way, by showing that treatment with SP600125 could significantly reduce cordycepin-induced DNA fragmentation, another indicator of apoptosis (Figure 5(b)). For an even stronger independent way to verify the role of JNK in cordycepin-induced apoptosis, we treated OEC-M1 cells with JNK6o, a more selective inhibitor of JNK than SP600125. Treatment with JNK6o also abolished the release of cytokeratin 18 fragments induced by cordycepin (Figure 5(c), *p* < 0.05). Furthermore, we reproduced the same results using the same treatments with cordycepin and SP600125 or JNK6o in OC3 cells, another oral cancer cell line derived from buccal epidermal carcinoma. We found that treatment with SP600125 or JNK6o both significantly inhibited the cordycepin-induced release of cytokeratin 18 fragments from OC3 cells (Figure 5(d), *p* < 0.05). Together, these data represent strong evidence that activation of JNK plays an essential role in cordycepin-induced apoptosis in oral cancer cells.

### 3.6. Cordycepin-Activated JNK Is Required for Activation of Caspase-8, Capase-9, and Caspase-3 and Cleavage of PARP

**Figure 6:**
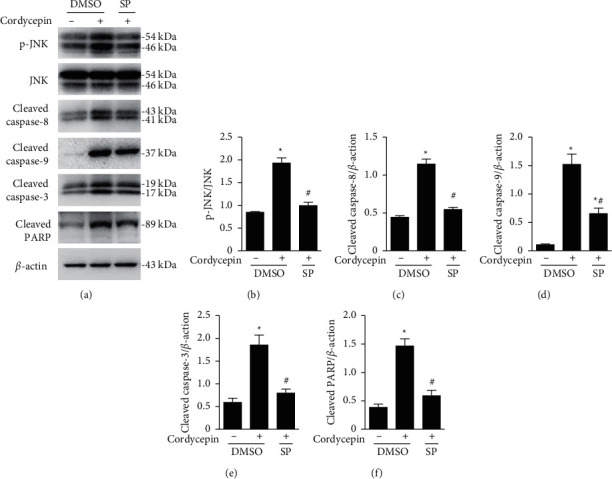
Cordycepin-activated JNK is required for the activation of caspase-8, caspase-9, and caspase-3 and the cleavage of PARP. OEC-M1 cells were treated without (-) or with 100 *μ*M cordycepin (+) and concurrently treated with 5 *μ*M SP600125 (SP), a JNK inhibitor, or the solvent control DMSO for 24 h. (a) Protein expression of phosphorylated JNK, total JNK, cleaved caspase-8, cleaved caspase-9, cleaved caspase-3, cleaved PARP, and *β*-actin (internal control) was examined using western blotting. Protein levels were quantified using the Quantity One image analysis system for (b) activated JNK, (c) cleaved caspase-8, (d) cleaved caspase-9, (e) cleaved caspase-3, and (f) cleaved PARP. ^*∗*^*p* < 0.05 vs. control + DMSO group; ^#^*p* < 0.05 vs. cordycepin + DMSO group.

We have shown that cordycepin-activated caspases (Figure 3), as well as JNK (Figure 4) and that JNK was required for cordycepin-induced apoptosis (Figure 5) in OEC-M1 cells. Given that JNK is involved in promoting caspase activation [26], we tested whether JNK is the upstream molecule of caspase activation induced by cordycepin by assaying the expression of activated proteins using western blotting (Figure 6(a)). The results of the quantitative analysis of the blots clearly demonstrated that SP600125 treatment reduced cordycepin-induced activation of JNK (Figure 6(b)) and the expression of cleaved caspase-8 (Figure 6(c)), cleaved caspase-9 (Figure 6(d)), cleaved caspase-3 (Figure 6(e)), and cleaved PARP (Figure 6(f)) (*p* < 0.05, for all). These results indicate that JNK is the upstream molecule of caspase-8, caspase-9, caspase-3, and PARP in OEC-M1 cells.

### 3.7. Summary Chart of the Molecular Pathways Implicated with Cordycepin-Induced Apoptosis in OEC-M1 Cells

**Figure 7:**
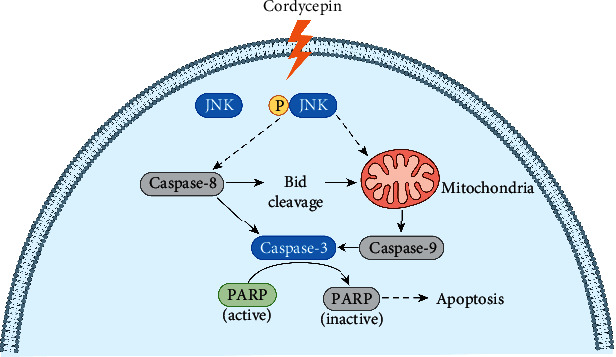
The signaling pathways implicated with cordycepin-induced apoptosis in OEC-M1 cells. The cordycepin-activated JNK plays a critical role in promoting the activation of caspases, which contributes to apoptosis in OEC-M1 cells.

To summarize the above results, we propose a hypothetical network of the signaling pathways as a model of the molecular mechanism underlying cordycepin-induced apoptosis in OEC-M1 cells (Figure 7). The cascade of activation in the network begins with cordycepin-activated JNK, which contributes to caspase-8 and caspase-9 activations. Activation of caspase-8 may lead to Bid cleavage, contributing to activation of the mitochondrial pathway. Activation of both caspase-8 and caspase-9 results in activation of caspase-3, which promotes PARP cleavage and, ultimately, to apoptosis in OEC-M1 cells.

## 4. Discussion

We used controlled protein expression analysis to explore the molecular basis of cordycepin-induced apoptosis in oral cancer cell cultures. First, we showed that Z-VAD-FMK (a pan-caspase inhibitor) effectively inhibited cordycepin-induced apoptosis (Figure 1), indicating that cordycepin induces apoptosis through activating caspases in OEC-M1 cells. Second, we showed that cordycepin treatment induced caspase-8 and caspase-9 activation in these cells (Figure 2), strongly suggesting that cordycepin may induce apoptosis through both the external and internal apoptosis pathways in OEC-M1 cells. Third, we showed that cordycepin treatment leads to the activation (cleaving) of Bid (Figure 3), a proapoptotic Bcl-2 protein. Since the Bid is cleaved by caspase-8, contributing to mitochondrial pathway activation [22], we speculate that the death receptor may connect with the mitochondrial pathway to induce cell apoptosis under cordycepin treatment in OEC-M1 cells. Fourth, we showed that the activation of JNK was essential for cordycepin-induced apoptosis in OEC-M1 cells. While cordycepin-activated many MAPKs, including JNK, ERK, and p38 MAPK (Figure 4), only the inhibition of JNK (by SP600125), but not the other MAPKs, could effectively inhibit cordycepin-induced apoptosis (Figure 5). This finding is consistent with previous studies, which also showed that JNK plays a vital role in mediating apoptosis in lung cancer cells and ovarian cancer cells, respectively [30, 31]. Fifth, we showed that in cells where JNK was inhibited, the activation of caspase-8 and caspase-9 was also inhibited (Figure 6), placing JNK to the upstream in the cascade of caspase-8 and caspase-9 activation in OEC-M1 cells. However, it remains elusive how JNK activates the initiator caspase-8 and caspase-9 under cordycepin treatment in OEC-M1 cells.

It has been demonstrated that the death receptor pathway is activated when Fas Ligand (FasL), a death factor, binds its receptor, Fas [32]. Since JNK reportedly promotes the expression of FasL [33], it is possible that JNK activates caspase-8 through the FasL/Fas death receptor pathway to induce cancer cell apoptosis. On the other hand, JNK could induce cancer cell apoptosis by activating proapoptotic Bcl-2 proteins Bax and Bim [34, 35] that contribute to caspase-9 activation in the mitochondrial pathway [22, 36]. Thus, we speculate that cordycepin could stimulate JNK, which would then simultaneously activate both the death receptor pathway and the mitochondrial pathway to induce OEC-M1 cell apoptosis.

There is evidence that the ERK pathway, in addition to promoting apoptosis, might have a role associated with cell survival [37]. In fact, we found that ERK inhibition significantly enhanced cordycepin-induced apoptosis in OEC-M1 cells, in sharp contrast to the effect of JNK inhibition (Figure 5(a)), consistent with the ERK function serving as a survival pathway during cordycepin treatment. In fact, studies have shown that treatment with ERK inhibitors could enhance tamoxifen-induced apoptosis and crizotinib-inhibited tumor growth [38, 39]. Together, these lines of evidence suggest that blocking ERK signaling could be an excellent strategy for enhancing cordycepin-induced apoptosis in OEC-M1 cells, on the condition that targeted future *in vitro* and *in vivo* experiments will corroborate this scenario.

The tumor suppressor p53 gene is important in the regulation of apoptosis [40], promoting the activation of Bid and caspases [41, 42] or upregulating the expression of proapoptosis-related proteins such as Bax and PUMA [43, 44]. Previously, it was shown that JNK activated p53 to promote apoptosis [45]. However, we showed that cordycepin promoted the activation of caspases (Figure 2) and Bid (Figure 3) to induce apoptosis in OEC-M1 cells, a p53 mutation cell line, discounting the scenario that cordycepin-induced apoptosis depended on the p53 pathway.

The molecular pathway through which cordycepin induces the activation of JNK in OEC-M1 cells remains to be elucidated. One study has reported that cordycepin promoted the production of reactive oxygen species (ROS) [46], which is a factor known to induce the activation of apoptosis signal-regulating kinase 1 (ASK1), an upstream molecule of JNK [47]. In an alternative scenario, cordycepin could activate AMP-activated protein kinase (AMPK) [48], which is also known to promote the activation of JNK [49]. Thus, under a highly possible scenario, cordycepin activates JNK by activating the ROS-ASK1 and/or AMPK pathways to induce apoptosis in OEC-M1 cells, but further studies are necessary to work out the details of this mechanism.

## 5. Conclusion

Cordycepin activates JNK and the caspase pathways to induce apoptosis in OEC-M1 cells. These results can be used to design more successful therapy regimes combined with cordycepin anticancer effects to improve the prognosis of patients with oral cancer.

## Data Availability

The data used and/or analyzed in the present study are available from the corresponding authors upon request.
